# A Novel Compound from the Mushroom *Cryptoporus volvatus* Inhibits Porcine Reproductive and Respiratory Syndrome Virus (PRRSV) *In Vitro*

**DOI:** 10.1371/journal.pone.0079333

**Published:** 2013-11-18

**Authors:** Zengqiang Ma, Weiwei Zhang, Li Wang, Mengjuan Zhu, Hexiang Wang, Wen-hai Feng, Tzi Bun Ng

**Affiliations:** 1 State Key Laboratory for Agrobiotechnology and Department of Microbiology, China Agricultural University, Beijing, China; 2 School of Biomedical Sciences, Faculty of Medicine, The Chinese University of Hong Kong, Hong Kong, China; Virginia Polytechnic Institute and State University, United States of America

## Abstract

Porcine reproductive and respiratory syndrome (PRRS), caused by PRRS virus (PRRSV), is a serious contagious disease in the swine industry. At present, there are no effective control strategies against PRRSV. Thus, there is an urgent need for new treatment regimens that have efficacious antiviral activity to compensate for vaccines. The anti-infective effect of *Cryptoporus volvatus* has previously been demonstrated in Tradational Chinese Medicine. In this report, we expected to identify a new anti-PRRSV agent in the aqueous extract of *C. volvatus*, by employing a combination of modern chromatographic purification techniques and indirect immunofluorescence assay (IFA). Our results showed that *C. volvatus* extracts from every separation step differed in their inhibitory potency on PRRSV. One anti-PRRSV component designated as C_M-H-L-5_ was isolated from water-soluble fraction of *C. volvatus*. The inhibition induced by C_M-H-L-5_ occurred in a dose-dependent manner. C_M-H-L-5_ appeared to be a low-molecular-weight polyol fragment with amide groups and carboxylic acid groups. Collectively, our findings imply that C_M-H-L-5_ from the aqueous extract of *C. volvatus* has the potential to be used for anti-PRRSV therapy.

## Introduction

Porcine reproductive and respiratory syndrome (PRRS) is a serious contagious disease in the swine industry, causing significant economical losses worldwide [1], [2]. The causative agent, PRRS virus (PRRSV), can cause reproductive failure in pregnant sows, respiratory diseases in piglets, and asymptomatic infections in boars [1]. Most recently, there were devastating outbreaks of atypical PRRS in China, which is characterized by high fever, high morbidity, and high mortality in pigs of all ages [3], [4]. The causative agent is a highly pathogenic PRRSV (HP-PRRSV) genotype with a discontinuous deletion of 30 amino acids in nonstructural protein 2 (nsp2) [3], [4], [5], [6]. PRRSV belongs to the family Arteriviridae of the order Nidovirales, and its genome is a single-stranded, positive-sense RNA [7], [8].

At present, vaccination is the prevailing way to control PRRS virus infection. However, commercial vaccines against PRRS virus have serious problems related to efficacy and safety. Antiviral therapeutics constitute a critical tool for combating viral infections, especially for cases in which there are no vaccines to match well with the circulating virus. Thus, an alternative measure to control PRRSV is pharmacological intervention. Previous studies have discovered a few natural compounds and compositions that have antiviral activities on PRRSV [9]. However, until now there are no effective commercial drugs available to control PRRSV infection. Currently used antiviral agents are often costly, have significant side effects [10], and lead to development of drug resistance in virus populations evolving under selective pressures [11], [12]. As a result, antiviral natural products are candidates to be developed as new generations of antivirals administered either alone or, preferably, in combination with current modalities [13].

The medical use of mushrooms has a long tradition in Asian countries, and their use in the Western hemisphere has increased slightly in the past decades [14], [15], [16], [17]. Whole extracts [18] and also isolated compounds [19], [20] of medicinal mushrooms have been shown to have antiviral effects. With advances in fractionation techniques for isolating and purifying natural products and in analytical techniques for structural determination, screening of natural product mixtures is now more compatible with the expected timescale of high-throughput screening campaigns. *C. volvatus* belongs to Aphyllophorales [21], and grows in certain areas of China. Its fruiting body had been used for the treatment of asthma and bronchitis since the 15th century AD. [22]. Aqueous extract from the fruiting body of *C. volvatus* has been reported to have polysaccharose, proteins, volatile oil, and cryptoporic acids, etc and anti-tumor, anti-allergy, anti-inflammatory, and immunomodulatory activities [23], [24], [25].

Our research team had reported that the aqueous extract of the fruiting bodies of *C. volvatus* had the potential to be used for antiviral therapy [26]. However, further information about the antiviral principles in *C. volvatus* is unavailable. In order to identify a new antiviral agent, we set out to isolate and purify the active compounds from the aqueous extract of *C. volvatus* fruiting bodies.

## Materials and Methods

### Material

*C. volvatus* was purchased from a market in the Yunnan Province of China. The mushroom was authenticated and a voucher specimen was deposited in our laboratory.

### Extraction and Isolation

Air-dried fruiting bodies of *C. volvatus* (200 g) were pulverized in a grinder and extracted overnight with distilled water (1500 ml) at 4°C. After leaving in a water bath at 65°C for 1 h, the slurry was centrifuged at 9,000 g for 30 min. The residue was further extracted by incubation in a water bath in 500 ml distilled water for 1 h. The extraction process was repeated twice, and the combined supernatant, named C, was filtered through a Millipore ultrafiltration membrane (NMRWL: 1000, Millipore). The ultrafiltrate was evaporated to dryness to give the resulting crude extract C_M_. The crude extract C_M_ (1 g) was dissolved in 2 ml distilled water, and then further purified sequentially by macroporous resin column chromatography, anion exchange chromatography on DEAE-cellulose, and Sephadex LH-20 chromatography.

### Purification by Employing Macroporous Resino Column

The crude extract was applied to a nonpolar macroporous resin column (HP-20, 5.5×20 cm, Mitsubishi) and a polar macroporous resin column (HP-2MGL, 5.5×20 cm, Mitsubishi) successively. After unabsorbed material had been eluted with distilled water, the polar macroporous resino column was eluted with a gradient of 0 to 100% ethanol solution. Absorbance at 260 nm was measured. Active fractions were collected for lyophilization.

### Purification by Employing Anion Exchange Chromatography on DEAE-cellulose

The resulting active fractions of the aqueous extract of *C. volvatus* were further dissolved in 10 mM NH_4_HCO_3_ buffer (pH 9.4) before centrifugation to remove insoluble material. The solution was chromatographed on a 2.5 cm×35 cm column of DEAE-cellulose (Sigma) which had been equilibrated with and was then eluted with 10 mM NH_4_HCO_3_ buffer (pH 9.4). The active component was collected and evaporated to dryness.

### Purification by Employing Sephadex LH-20 Chromatography

The active fractions were further fractionated by size-exclusion chromatography on Sephadex LH-20 (Pharmacia) [27]. Sephadex LH-20 was swollen in 20% ethanol overnight and then packed in a C10/20 column. Active fractions were chromatographed on the Sephadex LH-20 column and eluted successively with distilled water, 30% ethanol, 60% ethanol, 50% acetone at a flow rate of 0.2 ml/min. The eluate was monitored at UV 260 nm and 4-ml fractions were collected. Active fractions were collected and then dried by using a freeze-dryer (FD5-4, SIM). PRRSV inhibitory activity was checked by fluorogenic assays as previously described [26]. The fractions with the highest activity were further analysed by reverse-phase HPLC.

### Reverse Phase HPLC Analysis and Purification

Chromatographically pure methanol and ultrapure water were filtered with a 0.45- µm filter membrane before degassing. The active fraction (1 mg or 10 mg) from Sephadex LH-20 chromatography was dissolved in 1 ml of absolute methanol. Before analysis by HPLC coupled to PDA, the sample was filtered through a 0.45-µm nylon filter. Separation was achieved on an Agilent reverse phase C18 column (analytical column or preparation column) at 25°C. Elution was performed with H_2_O-MeOH (95∶5, vol:vol) using a flow rate of 1 ml/min or 3 ml/min. Detection was carried out in PDA, using 260 nm as wavelength. The combined active fractions were evaporated to dryness to give the extracts.

### NMR, MS Spectra and Detection of Major Chemical Functional Groups

ESI-FT-ICR-MS spectra were obtained by using an Acuu TOF CS, and ^1^H- and ^13^C-NMR spectra were acquired with a BRUKER AVANCEIII-400 spectrometer.

The chemical color reactions were carried out on TLC silica gel (2.5 cm×8 cm, EMD Millipore) with Dragendorff, Ninhydrin and Bromophenol Blue (BPB) reagents.

### Cells, Viruses, and Virus Preparation

Marc-145 cells are a PRRSV-permissive cell line sub-cloned from MA-104 cells [28]. Marc-145 cells were maintained in Dulbecco’s minimum essential medium (DMEM) supplemented with 10% FBS and penicillin/streptomycin. Porcine alveolar macrophages (PAMs) were obtained by postmortem lung lavage of 8-week-old specific pathogen free (SPF) pigs, and maintained in RPMI 1640 supplemented with 10% FBS and penicillin/streptomycin.

PRRSV strains, CH-1a (the first type 2 PRRSV strain isolated in China), VR2332 (the prototype of Type 2 PRRSV strain), and HV (a highly pathogenic PRRSV (HP-PRRSV) isolate, GenBank accession no. JX317648), were propagated in Marc-145 cells or PAMs. Virus preparations were titrated on Marc-145 cells or PAMs, and then stored at -80°C. Briefly, PRRSV was serially diluted 10-fold in complete DMEM or RPMI1640 to infect 5×10^4^ Marc-145 cells or PAMs in 96-well plates. PRRSV infection was determined 72 h post infection using immunofluorescent staining for the PRRSV N protein. Virus titer was determined using Reed-Muench method, and expressed as tissue culture infective dose 50% (TCID_50_). PFU was determined according to ‘‘PFU = 0.7*TCID_50_’’ as described before [29], and the multiplicity of infection (MOI) was calculated based on PFU.

### Assay of Inhibition of Virus Infection

Marc-145 cells or PAMs in 96-well plates were inoculated with Ch-1a, HV or VR2332 at an MOI of 0.1 for 2 h at 37°C, and then the viral inoculum was removed and fresh medium containing 2% FBS and different concentrations of the *Cryptoporus volvatus* extract or IFN-a (10 units/ml), a known inhibitor of PRRSV replication [30], was added. Twenty-four hours later, the supernatant was collected for virus titration, and cells were fixed for indirect immunofluorescence assay. The 50% effective concentration (EC_50_) was determined using a 4 parameter, nonlinear regression of dose response inhibition by plotting log (inhibitor(-concentration)) vs. virus titer (variable slope) using GraphPad Prism (GraphPad Software, San Diego, CA).

### Indirect Immunofluorescence Assay (IFA)

Cells were fixed with cold methanol-acetone (1∶1, vol:vol) for 10 min at 4°C, washed with phosphate-buffered saline (PBS), and then blocked with 5% normal goat serum for 30 min at 37°C. After blocking, cells were stained with anti-PRRSV N protein monoclonal antibody SDOW17 (1∶10,000, Rural Technologies) for 60 min at room temperature. Cells were then washed and incubated with FITC-conjugated goat anti-mouse IgG (H+L) (1∶2000, Jackson ImmunoResearch) for 1 h at 37°C. After three washes in PBS, cells were counter-stained with DAPI and examined by fluorescence microscopy.

### MTT Assay

The MTT [3-(4,5-dimethyl-2-thiazolyl)-2,5-diphenyl-2H-tet-razo-lium bromide] assay was used to examine the effect of the *Cryptoporus volvatus* extract on cell viability. Marc-145 cells or PAMs in 96-well plates were treated with sequential dilutions of the extract or normal saline in a total of 100 ml growth medium for 48 h. And then, 20 ml of freshly made 5 mg/ml MTT solution was added to each well, and the cells were incubated at 37°C for another 5 h before the medium was replaced with 200 ml DMSO to dissolve the crystals. The plates were further incubated at 37°C for 5 min to dissolve any air bubbles before absorbance due to the MTT signal was measured at 550 nm. The 50% cytotoxic concentration (CC_50_) was analyzed by GraphPad Prism (Graph-Pad Software, San Diego, CA).

### Real-time Reverse-transcription PCR (RT-PCR)

Total RNA was extracted from PAMs using the TRIzol reagent. RNAs were converted to cDNA using Superscript III Reverse Transcriptase (Invitrogen). In replication assay, PRRSV RNA was detected using quantitative real-time RT-PCR with primers designed against PRRSV ORF7 [31]. A plasmid containing PRRSV ORF7 sequence was used to generate a standard curve [32], and then RNA copies in all samples were calculated by comparing with it. For the transcript levels of cytokines, relative expressions of IFN-α, IFN-β, IL1-β and TNF-α in C_M-H-L-5_-treated or non-treated PAMs with or without PRRSV (HV strain) infection were quantified by the 2^−ΔΔCT^ Method [33]. GAPDH (glyceraldehyde-3-phosphate dehydrogenase) mRNA was set as a control. The primers used for real-time PCR amplification were listed in Table 1.

**Table 1:** List of primers for real-time PCR.

Name^a^	Sequences (50-30)^b^
ORF7-F1	AATAACAACGGCAAGCAGCA
ORF7-R1	GCACAGTATGATGCGTCGGC
IFN-α-F1	AGAGCCTCCTGCACCAGTTCT
IFN-α-R1	CTGCATGACACAGGCTTCCA
IL-1β-F1	TCTGCCCTGTACCCCAACTG
IL-1β-R1	CCCAGGAAGACGGGCTTT
IFN-β-F1	AGCACTGGCTGGAATGAAACCG
IFN-β-R1	CTCCAGGTCATCCATCTGCCCA
GAPDH-F1	CCTTCCGTGTCCCTACTGCCAAC
GAPDH-R1	GACGCCTGCTTCACCACCTTCT
TNF-α-F1 TNF-α-R1	CCCCCAGAAGGAAGAGTTTC CGGGCTTATCTGAGGTTTGA

a F1: forward primer, R1: reverse primer.

b Swine gene sequences were downloaded from GenBank.

### Statistical Analysis

All experiments were performed with at least three independent replicates. Results were analyzed using Student’s t test. Differences were considered to be statistically significant if the P value is less than 0.05. *P<0.05; **P<0.01; ***P<0.001.

## Results

### Chromatographic Action of Aqueous Extract from *C. Volvatus*

**Figure 1:**
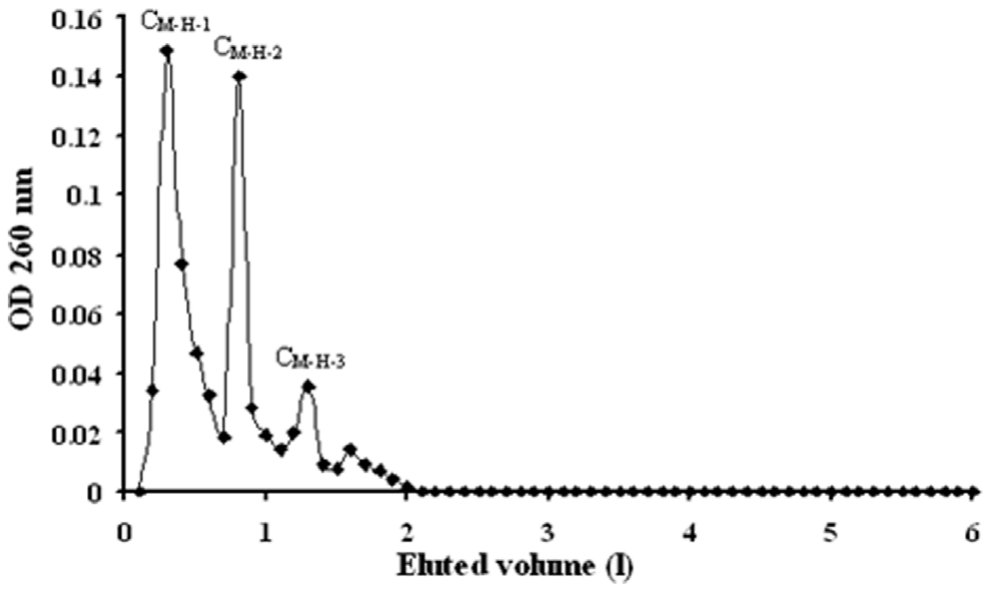
Elution profile of crude extract C_M_ from HP-2MGL macroporous resino column. Fraction collected through elution with 2% ethanol, corresponding to the strand on the x axis between 0 and 2 l; fraction collected through elution with 2 l of 60% ethanol, corresponding to the strand on the x axis between 2 and 4 l; and fraction collected through sample elution with 2 l of 100% ethanol, corresponding to the strand on the x axis between 4 and 6 l. The ultraviolet absorption of C_M-H_ contained three inner peaks, C_M-H-1_, C_M-H-2_ and C_M-H-3_.

The crude extract C_M_ was first chromatographed on HP-20 to remove impurities, and then the unadsorbed components were loaded onto HP-2MGL. The components adsorbed on HP-2MGL were eluted with an ethanol gradient (30%, 60% and 100% ethanol). The elution profile of the fractions from HP-2MGL is shown in Fig. 1. Three fractions were obtained from the HP-2MGL chromatography, corresponding to 30%, 60% and 100% ethanol eluate respectively. The fraction eluted with 30% ethanol was designated as C_M-H._ The fractions eluted with 60% and 100% ethanol were not named (Fig. 1). Besides, the ultraviolet absorption of C_M-H_ was higher than any other fraction (fraction eluted with 60% or 100% ethanol), and showed several inner peaks, C_M-H-1_,

### C_M-H-2_ and C_M-H-3_, as indicated by OD 260 nm

**Figure 2:**
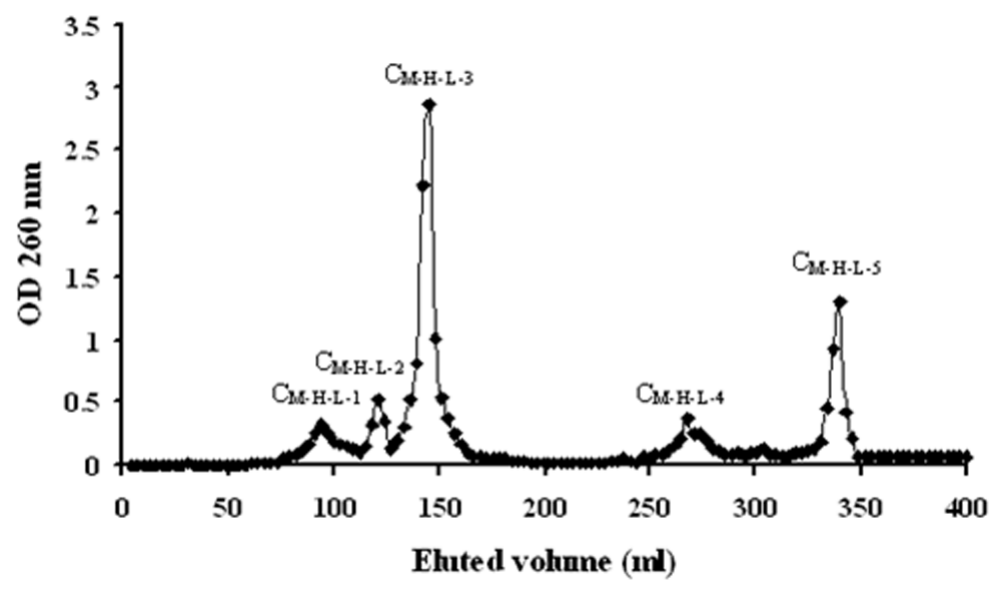
Elution profile of crude extract C_M-H-L_ from Sephadex LH-20. Fraction collected through elution with 100% ethanol, 100 ml of 60% ethanol, 100 ml of 100% ethanol, corresponding to the strand on the x axis between 0 and 100 ml, 100 and 200 ml, 200 and 300 ml, 300 and 400 ml respectively. Sephadex LH-20 yielded five fractions at OD 260 nm, C_M-H-L-1_, C_M-H-L-2_, C_M-H-L-3_, C_M-H-L-4_ and C_M-H-L-5_.

The fraction C_M-H_ was loaded on a DEAE-cellulose ion exchange chromatography column, and the unadsorbed fraction was collected. This fraction was selected for size exclusion chromatography on Sephadex LH-20 (Fig. 2). Five fractions resulted, C_M-H-L-1_, C_M-H-L-2_, C_M-H-L-3_, C_M-H-L-4_, and C_M-H-L-5_. C_M-H-L-3_ was a filemot powder eluted from Sephadex LH-20 as a single, sharp peak. C_M-H-L-5_ was a white powder eluted from Sephadex LH-20 as a relative broad, small single peak.

We isolated and purified several fractions, C, C_M_, C_M-H_, and C_M-H-L-5_, from the fruiting body of *C. volvatus*.

### C_M-H-L-5_ Inhibits PRRSV Replication

**Figure 3:**
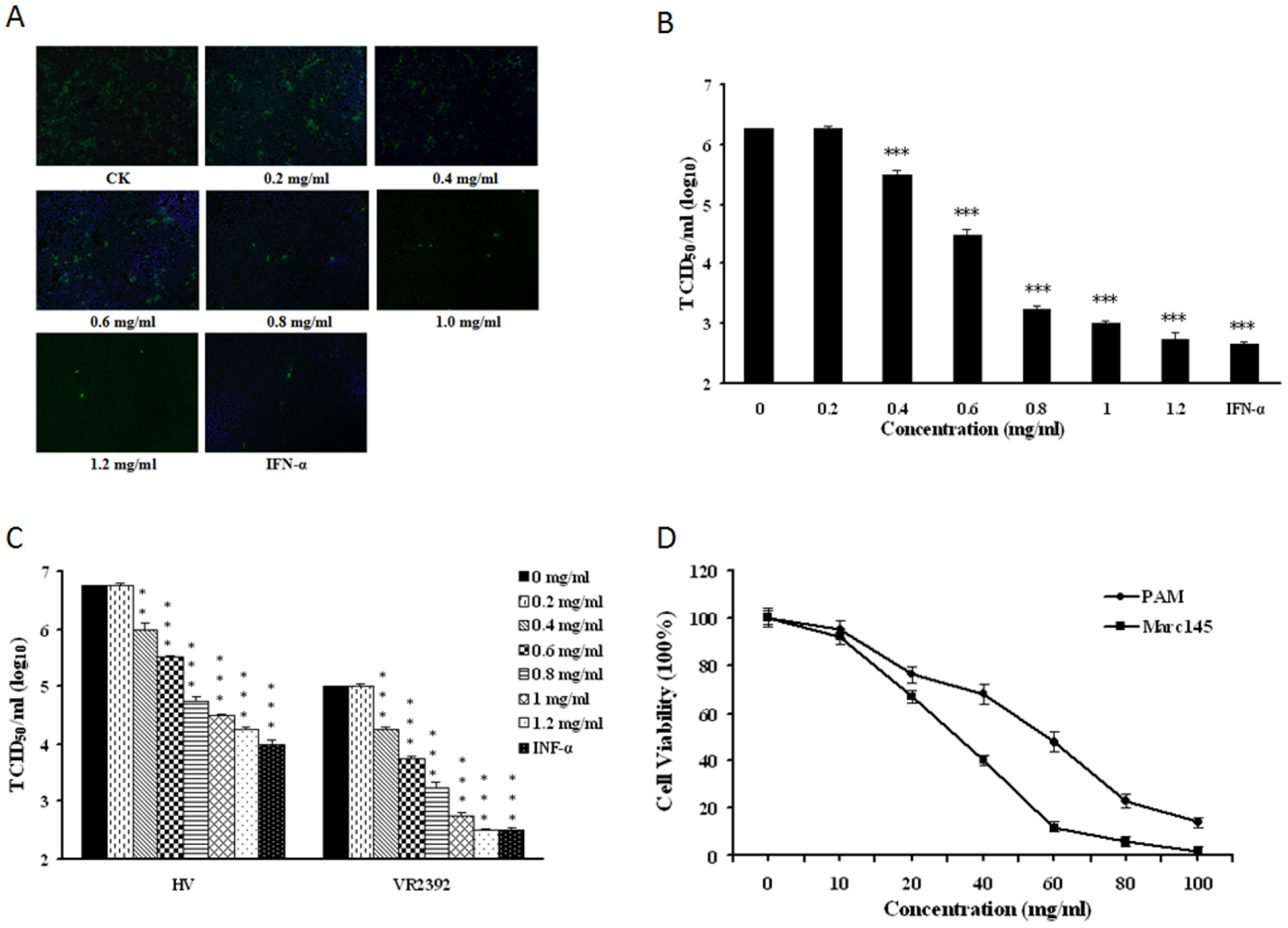
*C. volvatus* fraction C_M-H-L-5_ inhibited PRRSV replication. (A and B) The C_M-H-L-5_ blocked PRRSV Ch1a replication in Marc-145 cells. Marc-145 cells were infected with PRRSV Ch1a at an MOI of 0.1, and then treated with IFN-a (10 units/µl) or the C_M-H-L-5_ at various concentrations. At 24 h p.i., cells were fixed and analyzed by IFA using antibody against PRRSV N protein (A), and virus yield in the supernatants was also quantified (B). Culture treated with normal saline was set up as negative control (0 mg/ml), and treated with INF-α was set up as positive control. The brightness of the fluorescence represents the level of the virus. Results are from three independent experiments, each of which was conducted in triplicate. (C) The C_M-H-L-5_ potently inhibited both PRRSV HV and VR2332 replication in PAMs. A similar virus inhibition assay was performed with PAM cells infected with PRRSV strain HV or VR2332 at an MOI of 0.1 in the presence of IFN-a (10 units/µl) or the C_M-H-L-5_ at various concentrations. (D) Determination of cytotoxicity of the C_M-H-L-5_ by MTT assay. PAMs or Marc145 cells were incubated with various concentrations of the C_M-H-L-5_ or the control normal saline for 48 h prior to the MTT assay. Data are representative of three independent experiments (mean ± SD). Statistical significance was analyzed by Student’s t test. *P<0.05; **P<0.01; ***P<0.001.

To explore the antiviral activity of C_M-H-L-5_ against viruses, we first investigated its antiviral effect on PRRSV infection. The antiviral activity of related fraction was tested by IFA and the brightness of the fluorescence which represented the level of the virus. As shown in Fig. 3A and B, C_M-H-L-5_ significantly inhibited PRRSV (CH-1a strain) replication in Marc-145cells. The extract at the concentration of 1.2 mg/ml reduced the virus yields by about 3000-fold when compared to normal saline control, and this inhibition was in a dose-dependent manner. To further verify its anti-PRRSV activity, we examined whether C_M-H-L-5_ could inhibit replication of more than one PRRSV strain in PAMs. As illustrated in Fig. 3C, C_M-H-L-5_ potently inhibited the replication of both the prototype of Type 2 PRRSV strain (VR2332) and HP-PRRSV strain (HV) in PAMs, which could reach a 300-fold suppression at the concentration of 1.2 mg/ml. The extract inhibited PRRSV infection with a 50% effective concentration (EC_50_) value of 0.29 mg/ml for CH-1a strain in Marc-145 cells, 0.29 mg/ml for HV strain, and 0.31 mg/ml for VR2332 in PAMs.

To exclude the possibility that nonspecific toxicity induced by the extract could affect PRRSV replication, we evaluated PAM and Marc-145 cell viability under various concentrations of C_M-H-L-5_ using the MTT assay (Fig. 3D). The 50% cytotoxic concentrations (CC_50_) of the C_M-H-L-5_ for PAMs and Marc-145 cells were 55 mg/ml and 28 mg/ml, respectively, which greatly exceeded its EC_50_ value. The therapeutic index (CC_50_/EC_50_) was 97 for CH-1a strain in Marc-145 cells, 190 for HV strain, and 177 for VR2332 in PAMs.

These initial studies confirmed that the *C. volvatus* extract C_M-H-L-5_ could inhibit PRRSV infection. Therefore, it is necessary for us to compare antiviral cytokine gene expression and different *C. volvatus* extracts on inhibition of PRRSV in subsequent studies.

### Antiviral Cytokine Gene Expression in C_M-H-L-5_-treated Porcine Alveolar Macrophages

**Figure 4:**
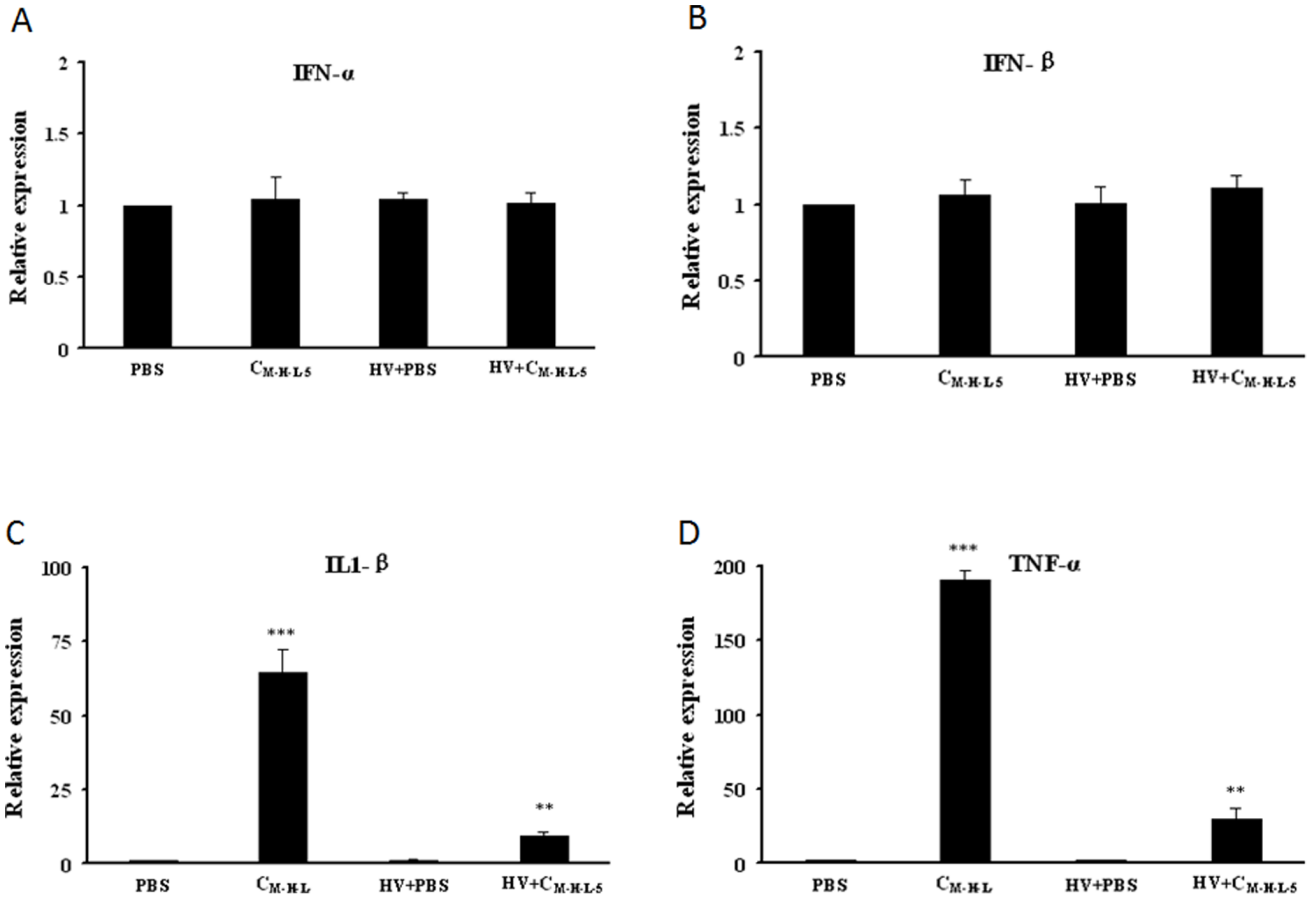
Transcript levels of cytokines in PAMs. PAMs were infected with HV at an MOI of 0.1 in the presence or absence of C_M-H-L-5_. Expressions of IFN-α (A), IFN-β (B), IL1-β (C)and TNF-α (D) were analyzed using Real-time RT-PCR at 12 h after treated with HV, HV plus C_M-H-L-5_ (0.8 mg/ml), or C_M-H-L-5_ only. Relative expression (fold changes) in comparison with PBS (Phosphate buffer saline) -treated cells (set up as 1) is shown. The data represent the mean ± standard deviation from three independent experiments. Significant differences between groups treated with C_M-H-L-5_ and for PBS PRRSV infected cells or uninfected cells are denoted by ‘‘*’’ (P<0.05), ‘‘**’’ (P<0.01), and “***” (P<0.001).

Cytokines are able to interfere with viral infection. Thus, we postulated that C_M-H-L-5_ might induce antiviral cytokine expressions. To investigate this possibility, the expressions of four cytokines including IFN-α, IFN-β, IL1-β and TNF-α, known to be involved in antiviral response and inflammation, were analyzed in the presence or absence of C_M-H-L-5_. Porcine alveolar macrophages (PAMs) were incubated with HV, HV plus C_M-H-L-5_ (0.8 mg/ml), or C_M-H-L-5_ only, and real-time RT-PCR was performed to assess the relative mRNA level in PAMs after cultured for 12 h. C_M-H-L-5_ did not significantly induce IFN-α (Fig. 4A) and IFN-β (Fig. 4B) expression in both infected and non-infected PAMs. However, C_M-H-L-5_ treatment could elevate the levels of IL-1β (Fig. 4C) and TNF-α (Fig. 4D) expression in PAMs. The expressions of IL1-β and TNF-α exhibited 8-fold and 25-fold increase in C_M-H-L-5_-treated infected cells respectively. Taken together, these data suggested that C_M-H-L-5_-impaired PRRSV infection could be partially due to the up-regulation of certain cytokines.

### Effect of Different Extracts on Inhibition of PRRSV

**Figure 5:**
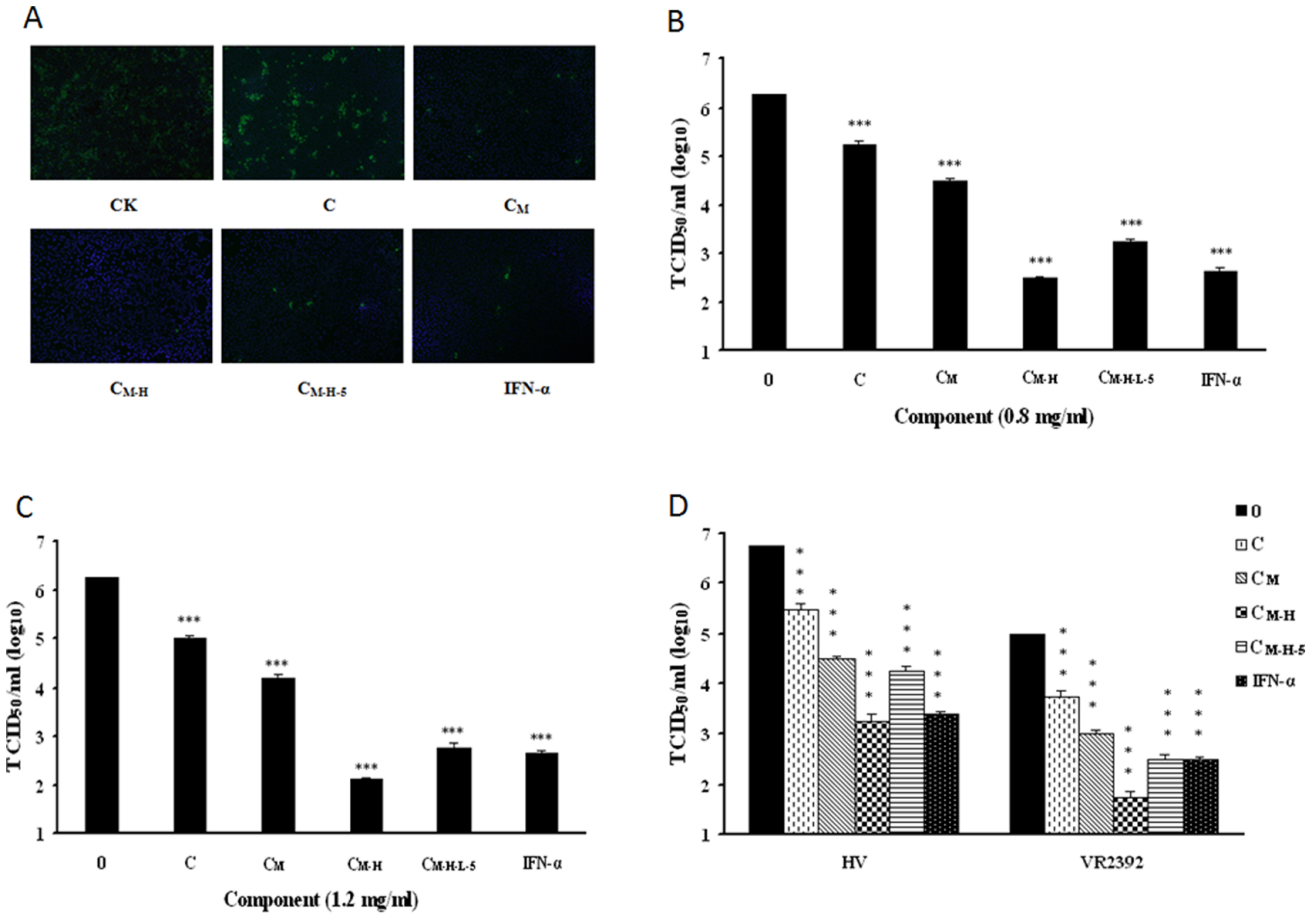
Different *C. volvatus* fractions blocked PRRSV replication in Marc-145 cells and PAMs. (A, B and C) Marc-145 cells were infected with PRRSV Ch1a at an MOI of 0.1, and then treated with different *C. volvatus* extracts at a concentration of 0.8 mg/ml or INF-α (10 units/µl). At 24 h post-infection, cells were fixed and analysed by IFA using antibody against PRRSV N protein (A). The abbreviations at the bottom of fluorescent pictures are explained as follows: **C**: Aqueous extract of *C. volvatus*; **C_M_**: C was filtered through a Millipore ultrafiltration membrane and the ultrafiltrate gave the resulting crude extract C_M_; **C_M-H_**: C_M_ absorbed on HP-2MGL was eluted with 30% ethanol, which resulted in C_M-H_; **C_M-H-L-5_**: C_M-H_ was loaded on a DEAE and Sephadex LH-20 chromatography column sequentially, which resulted in C_M-H-L-5_. Virus yield in the supernatant was quantified (B). The same experiment was carried out with different *C. volvatus* extracts at a concentration of 1.2 mg/ml or INF-α (10 units/µl) (C). (D) Different *C. volvatus* fractions inhibited replication of VR2332 and HV strains in PAMs at a concentration of 1.2 mg/ml or INF-α (10 units/µl). Virus yield in the supernatant was also quantified. Culture treated with normal saline was set up as negative control (0 mg/ml), and that treated with INF-α was set up as positive control. The brightness of the fluorescence represents the level of the virus. Data are representative of three independent experiments (mean ± SD). Statistical significance was analyzed by Student’s t test. *P<0.05; **P<0.01; ***P<0.001.

Next, we investigated the effect of C, C_M_, C_M-H_, and C_M-H-L-5_ on PRRSV infection to compare the antiviral activity of different *C. volvatus* extracts against PRRSV viruses. As shown in Fig. 5A and B, C, C_M_, C_M-H_ and C_M-H-L-5_ significantly inhibited PRRSV (CH-1a strain) replication in Marc-145 cells which produced different extents of inhibition of PRRSV at a concentration of 0.8 mg/ml. As the purification proceeded, the virus titration decreased to the lowest in C_M-H_ treatment, and then significantly increased following C_M-H-L-5_ treatment. C_M-H_ elicited the strongest inhibition compared with other components. The virus titration values of C_M-H_ and C_M-H-L-5_ at a concentration of 0.8 mg/ml were 10^2.5^ TCID_50_/ml and 10^3.25^ TCID_50_/ml, respectively. The same trend occurred in treatment at 1.2 mg**/**ml, and the virus titration values of C_M-H_ and C_M-H-L-5_ were 10^2.1^ TCID_50_/ml and 10^2.75^ TCID_50_/ml, respectively (Fig. 5C). We further verified different *C. volvatus* extracts could inhibit replication of VR2332 and HV strains in PAMs in the same trends at a concentration of 1.2 mg/ml (Fig. 5D).

Taken together, these data showed that C, C_M_, C_M-H_, and C_M-H-L-5_ produced different extents of inhibition of PRRSV.

### Reverse Phase HPLC Analysis, Purification and Identification

**Figure 6:**
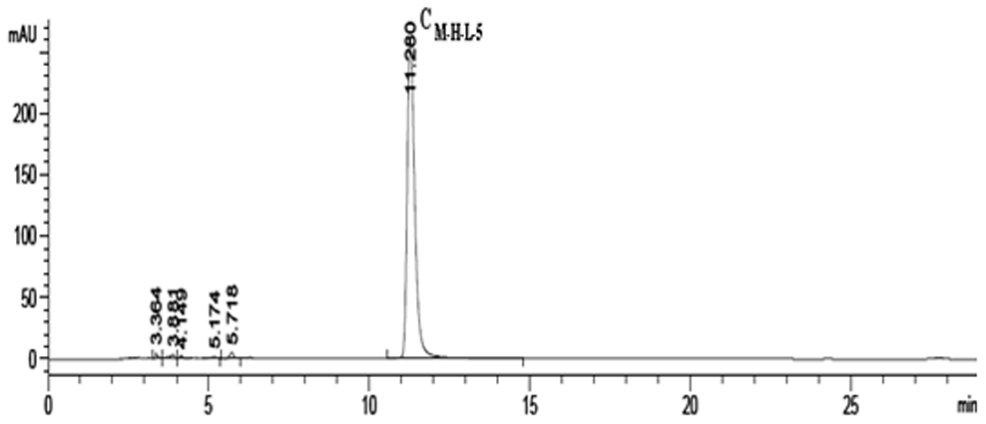
HPLC Chromatogram of *C. volvatus* fraction C_M-H-L-5_ at 260 nm. (mobile phase: methanol:water = 0.5∶95 (vol:vol), flow rate: 1 ml/min; temperature: 25°C, retention time: 11.280 min) C_M-H-L-5_ yielded a main, sharp peak when subjected to analytical HPLC, and the purity of C_M-H-L-5_ was 97%.

**Figure 7:**
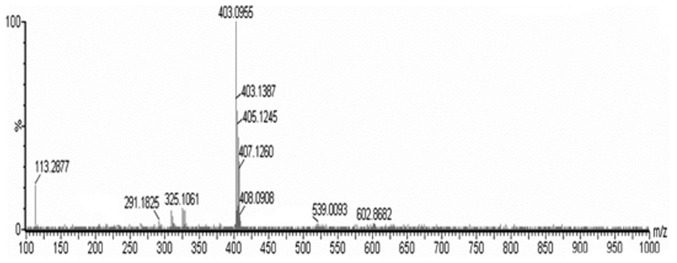
Mass spectrometric detection of *C. volvatus* chromatographic fraction C_M-H-L-5_. The HPLC-MS of C_M-H-L-5_ gave m/z data as the protonated molecular ion [M+H]^+^. The data of m/z of protonated molecular ion was deducted one, and then presented a molecular mass.

Fraction C_M-H-L-5_ was analysed or purified on an HPLC preparative column followed by an analytical column or a preparative column with 5% methanol. C_M-H-L-5_ yielded a single, sharp peak when subjected to analytical HPLC, and the purity of C_M-H-L-5_ was 97%. Currently, the retention time of C_M-H-L-5_ was 11.28 min (Fig. 6). Moreover, mass spectrometric analyses tested with electrospray ionization mass spectrometry (ESI-MS) showed that the m/z [M+H]^+^ of C_M-H-L-5_ was 404.0955 Da which presented a molecular mass as 403.1 Da (Fig. 7).

### ^1^H-NMR and ^13^C-NMR Spectra were Acquired with the Use of a BRUKER

AVANCEIII-400 spectrometer. The NMR spectrum showed that there were very little impurities in the sample (please refer to supplementary data). Based on nuclear magnetic resonance spectra data and spectrum information of structure analysis, it was deduced that the sample structure contained amide groups and carboxylic acid groups ; The peak at δ90-110 ppm in the ^13^C-NMR spectrum of C_M-H-L-5_ indicated the absence of sugar fragments. However, the peaks at δ60–70 ppm showed the presence of polyol fragment containing 11 or 12 carbon atoms (please refer to supplementary data).

**Figure 8:**
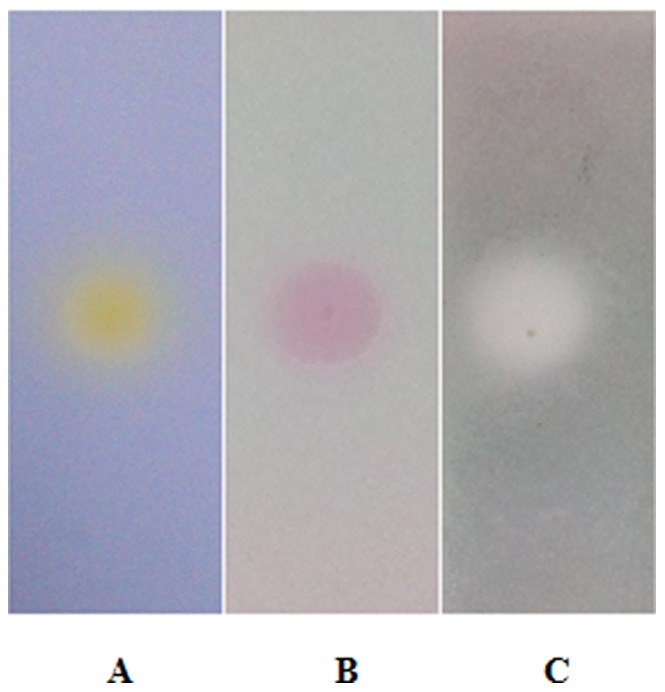
Detection of major chemical functional groups by color reactions. (A) Organic acid color reaction (0.05% BPB - ethanol solution): The BPB was used to detect the carboxylic acid group, along with yellow color change. (B) Ninhydrin color reaction: The existence of amide groups can induce mulberry color change in Ninhydrin color reaction. (C) Dragendorff color reaction: The compound, containing nitrogen atoms, can cause orange or yellow color change in Dragendorff color reaction. However, there are not any color changes when the nitrogen atoms are in the form of amide groups or not in existence.

The amide groups and carboxylic acid groups were further indicated by results of chemical color reactions (Dragendorff, Ninhydrin and Organic Acid Reaction). As shown in Fig. 8, the existence of amide groups induced mulberry color change in Ninhydrin color reaction and not any color changes in Dragendorff color reaction. Moreover, Bromophenol blue detected the carboxylic acid group, along with yellow color change.

Integrating the above structural information, it was concluded that C_M-H-L-5_ was a polyol fragment with amide groups and carboxylic acid groups.

## Discussion

*C. volvatus* is commonly used as an anti-infective agent in traditional Chinese medicine. The inhibitory effect of *C. volvatus* has been previously demonstrated. Gao et al. (2013) found that the aqueous extract from the fruiting bodies of *C. volvatus* has the potential to be used for antiviral therapy [26]. In the present study, we further isolated and purified the antiviral compounds in the aqueous extract prepared from the fruiting bodies of *C. volvatus*.

Three kinds of chromatographic techniques were used in this study to isolate C_M-H-L-5_ from *C. volvatus* in an efficient way [34]. Macroporous resin polymer contains a permanent network of pores, and the network is independent of the swelling state of the resin. The weak polar resin HP-2MGL with highly cross-linked structure is a good carrier which is appropriate for C_M-H-L-5_ with weak polarity. DEAE-cellulose is a weakly basic anion exchanger, which can adsorb the undesired electronegative components. Sephadex is a gel filtration medium prepared by cross-linking dextran with epichlorohydrin. Different types of Sephadex differ in their degree of cross-linking and modifying groups, and hence the degree of swelling and the molecular fractionation range. Sephadex LH-20 is a well-known gel filtration medium for removing contaminants for small biomolecules including C_M-H-L-5_ in a single step. Moreover, the sample was purified by using only distilled water and methanol which were safer than organic solvents such as chloroform, ethyl acetate and ether.

We also showed that C_M-H-L-5_ not only significantly inhibited CH-1a strain replication in Marc-145cells, but also VR2332 and HV strains in PAMs, excluding the possibility of nonspecific toxicity. All initial studies confirmed that C_M-H-L-5_ could inhibit PRRSV infection (Fig. 3). Besides, the decline in brightness of fluorescence represents the increasing antiviral effect of the fraction and a decline in the fluorescence brightness was associated with a lower virus titration. In our studies, the decline in fluorescence brightness and virus titration were observed with the increase of concentration, indicating that the inhibition of C_M-H-L-5_ was in a dose-dependent manner.

Our work provided evidences that C_M-H-L-5_ was able to inhibit viral replication *in vitro*. In subsequent studies, two cytokines, IL1-β and TNF-α, were confirmed to be induced by C_M-H-L-5_ treatment. This provided a possibility that C_M-H-L-5_ could indirectly inhibit the PRRSV replication by regulating some antiviral cytokines. However, more study is required to verify if the induced cytokines by C_M-H-L-5_ play a critical role in the C_M-H-L-5_-induced inhibition of PRRSV replication. Nevertheless, our data suggested that C_M-H-L-5_ is probably regulating the host immune response.

The aqueous extract from the fruiting bodies of *C. volvatus* is a crude extract, which has many components. The antiviral effects of the extract might result from a mixture of active compounds rather than from a single chemical entity, the different compounds in the mixture mediate a synergistic antiviral effect [26]. The efficacy of Traditional Chinese Medicine (TCM) is a characteristic of a complex mixture of chemical compounds present in the various herbs. The concept of combinatorial medicines has been exemplified by the drug cocktail used in the treatment of acquired immunodeficiency syndrome. *C. volvatus* fractions from every separation step differ in their PRRSV inhibitory potency. With the increase in purification, antiviral activity component first increased up to C_M-H_, and then decreased with C_M-H-L-5_ obviously. However, the antiviral activity of C_M-H-L-5_ was still much higher than that of the crude extract C, and C_M-H-L-5_ could achieve a 10^2^-fold suppression compared to crude extract C (Fig. 5). So we conclude that there are more than one active component exist in *C. volvatus*.

**Figure 9:**
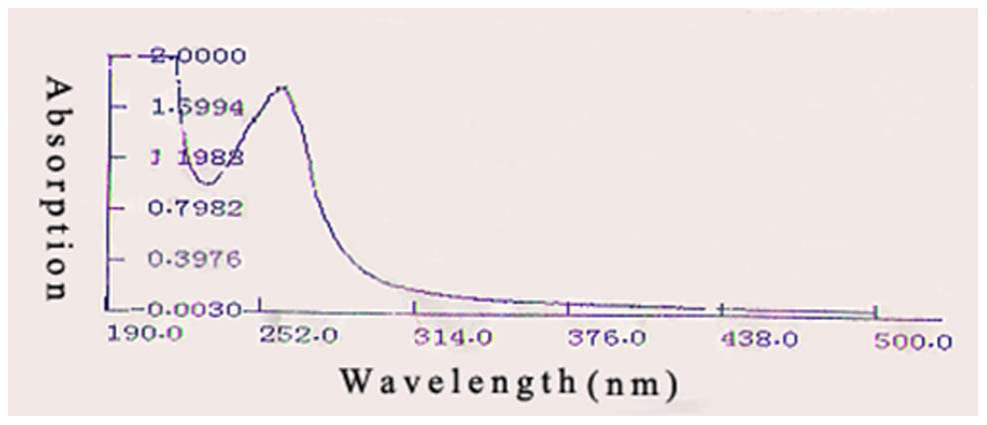
UV-VIS absorption spectrum of C_M-H-L-5_. (start wavelength: 190 nm, end wavelength: 500 nm). UV-VIS absorption spectrum scan showed that the value of ultraviolet absorbance of C_M-H-L-5_ at 260 nm was the highest.

As we know, the optimal wavelength of nucleoside absorbance is about 260 nm and the value of ultraviolet absorbance of C_M-H-L-5_ at 260 nm was the highest, which matched with the HPLC chromatogram and UV-VIS absorption spectrum scan (Figures 6 and 9). The amide groups not only exist in peptides, but also in several nucleosides, such as adenosine, guanosine, and uridine etc. Many antiviral nucleoside analogues derived from the aforementioned nucleosides have amide groups. Moreover, most antiviral drugs are nucleoside analogues which interfere with reverse transcriptase competitively [10]. Gao et al. proved that the crude extract of *C. volvatus* could interfere with reverse transcriptase for PRRSV inhibition [26]. As the most important antiviral component of *C. volvatus*, C_M-H-L-5_ may play an important role on reverse transcriptase inhibition. At last, we had isolated guanosine and uridine in similar separation methods with C_M-H-L-5_ for their similar physical properties. Those three substances may have similar structural features. Integrating the above information, it was concluded that C_M-H-L-5_ could be a nucleoside analogue or a polypeptide with a polyol fragment, and the nucleoside analogue or polypeptide component was composed of amino acids by condensation.

*C. volvatus* exerts a range of generally beneficial effects on respiratory disease and cancer. Cryptoporic polysaccharides and cryptoporic acids have been isolated from *C. volvatus* as significant active ingredients [35], [36], [37], [38]. Nowadays, most of the clinical antiviral drugs are nucleoside analogues [39]. In our study, C_M-H-L-5_ was first extracted from the edible mushroom *C. volvatus*. Besides, the structural nature of C_M-H-L-5_ was similar to both polysaccharides and nucleoside drugs, suggesting C_M-H-L-5_ might have a potential for medicinal use.

Above all, although the chemical composition of C_M-H-L-5_ of *C. volvatus* has been characterized, C_M-H-L-5_ is a novel antiviral compound with a very complex structure.

## Supporting Information

Figure S1 **^1^H-NMR spectrum.** ^1^H-NMR spectra were acquired with the use of a BRUKER AVANCEIII-400 spectrometer.
(TIF)

Figure S2 **^13^C-NMR spectrum (1).** ^13^C-NMR spectra were acquired with the use of a BRUKER AVANCEIII-600 spectrometer. The peaks at δ60–70 ppm showed the presence of polyol fragment containing 11 or 12 carbon atoms.
(TIF)

Figure S3 **^13^C-NMR spectrum (2).** ^13^C-NMR spectra were acquired with the use of a BRUKER AVANCEIII-600 spectrometer. The peak at δ90–110 ppm in the ^13^C-NMR spectrum of C_M-H-L-5_ indicated the absence of sugar fragments.
(TIF)

## References

[1] Neumann EJ, Kliebenstein JB, Johnson CD, Mabry JW, Bush EJ, et al (2005) Assessment of the economic impact of porcine reproductive and respiratory syndrome on swine production in the United States. J Am Vet Med Assoc 227(3): 385–392. 16121604 10.2460/javma.2005.227.385

[2] Tian KG, Yu XL, Zhao TZ, Feng YJ, Cao Z, et al (2007) Emergence of fatal PRRSV variants: unparalleled outbreaks of atypical PRRS in China and molecular dissection of the unique hallmark. PLoS One 2: e526. 17565379 10.1371/journal.pone.0000526 PMC1885284

[3] Li YF, Wang XL, Bo K, Wang XW, Tang B, et al (2007) Emergence of a highly pathogenic porcine reproductive and respiratory syndrome virus in the Mid-Eastern region of China. Vet J 174: 577–584. 17869553 10.1016/j.tvjl.2007.07.032

[4] Zhou YJ, Hao XF, Tian ZJ, Tong GZ, Yoo D, et al (2008) Highly virulent porcine reproductive and respiratory syndrome virus emerged in China. Transbound Emerg Dis 55: 152–164. 18405338 10.1111/j.1865-1682.2008.01020.x

[5] Ni JQ, Yang SB, Bounlom D, Yu XL, Zhou Z, et al (2012) Emergence and pathogenicity of highly pathogenic Porcine reproductive and respiratory syndrome virus in Vientiane, Lao People’s Democratic Republic. J Vet Diagn Invest 24: 349–354. 22379051 10.1177/1040638711434111

[6] Zhou L, Zhang JL, Zeng JW, Yin SY, Li YH, et al (2009) The 30-amino-acid deletion in the Nsp2 of highly pathogenic porcine reproductive and respiratory syndrome virus emerging in China is not related to its virulence. J Virol 83: 5156–5167. 19244318 10.1128/JVI.02678-08 PMC2682102

[7] Gorbalenya AE, Enjuanes L, Ziebuhr J, Snijder EJ (2006) Nidovirales: evolving the largest RNA virus genome. Virus Res 117: 17–37. 16503362 10.1016/j.virusres.2006.01.017 PMC7114179

[8] Snijder EJ, Meulenberg JJ (1998) The molecular biology of arteriviruses. J Gen Virol 79 (Pt 5): 961–979. 10.1099/0022-1317-79-5-961 9603311

[9] Karuppannan AK, Wu KX, Qiang J, Chu JJ, Kwang J (2012) Natural compounds inhibiting the replication of porcine reproductive and respiratory syndrome virus. Antiviral Res 94: 188–194. 22487208 10.1016/j.antiviral.2012.03.008 PMC7114079

[10] De Francesco R, Migliaccio G (2005) Challenges and successes in developing new therapies for hepatitis C. Nature. 436: 953–960. 10.1038/nature04080 16107835

[11] Yen HL, Hoffmann E, Taylor G, Scholtissek C, Monto AS, et al (2006) Importance of neuraminidase active-site residues to the neuraminidase inhibitor resistance of influenza viruses. J Virol 80: 8787–8795. 16912325 10.1128/JVI.00477-06 PMC1563878

[12] Rong L, Dahari H, Ribeiro RM, Perelson AS (2010) Rapid emergence of protease inhibitor resistance in hepatitis C virus. Sci Transl Med 2: 30ra32. 10.1126/scitranslmed.3000544 PMC3033690 20445200

[13] Harvey AL (2008) Natural products in drug discovery. Drug Discov Today 13: 894–901. 18691670 10.1016/j.drudis.2008.07.004

[14] Zjawiony JK (2004) Biologically active compounds from Aphyllophorales (polypore) fungi. J Nat Prod 67: 300–310. 14987072 10.1021/np030372w

[15] Stamets P (2006) Can mushrooms help save the world? Interview by Bonnie J. Horrigan. Explore (NY) 2: 152–161. 16781630 10.1016/j.explore.2005.12.011

[16] Wasser SP (2011) Current findings, future trends, and unsolved problems instudies of medicinal mushrooms. Appl Microbiol Biotechnol 89: 1323–1332. 21190105 10.1007/s00253-010-3067-4

[17] Lindequist U, Niedermeyer TH, Julich WD (2005) The pharmacological potential of mushrooms. Evid Based Complement Alternat Med 2: 285–299. 16136207 10.1093/ecam/neh107 PMC1193547

[18] Faccin LC, Benati F, Rincão VP, Mantovani MS, Soares SA, et al (2007) Antiviral activity of aqueous and ethanol extracts and of an isolated polysaccharide from *Agaricus brasiliensis* against poliovirus type 1. Lett Appl Microbiol 45: 24–28. 17594456 10.1111/j.1472-765X.2007.02153.x

[19] Mothana RAA, Awadh Ali NA, Jansen R, Wegner U, Mentel R, et al (2003) Antiviral lanostanoid triterpenes from the fungus *Ganoderma pfeifferi* . Fitoterapia 74: 177–180. 12628419 10.1016/s0367-326x(02)00305-2

[20] Yamamoto KA, Galhardi LCF, Rincão VP, Soares SA, et al (2013) Antiherpetic activity of an *Agaricus brasiliensis* polysaccharide, its sulfated derivative and fractions. Int J Biol Macromol 52C: 9–13. 10.1016/j.ijbiomac.2012.09.029 23043759

[21] Xu J (1997) Chinese medicinal mycology. Beijing: publishing house of Peking Union Medical College and China Medical University. 836 p.

[22] Wu ZY (1990) Xin-Hua Compendium of Materia Medica. Shanghai: Shanghai Science and Technology Publishing House. 735 p.

[23] Jin SH, Xie QM, Lin XX, Deng YM, Chen JQ (2003) Effect of *Cryptoporus volvatus* (Peck) Schear on leukotriene production from polymorphonuclear leukocytes in rats. Zhongguo Zhong Yao Za Zhi 28: 650–653. 15139113

[24] Yao HY, Zhang LH, Shen J, Shen HJ, Jia YL, et al (2011) Cyptoporus polysaccharide prevents lipopolysaccharide-induced acute lung injury associated with down-regulating Toll-like receptor 2 expression. J Ethnopharmacol 137: 1267–1274. 21875662 10.1016/j.jep.2011.07.058

[25] Xie QM, Deng JF, Deng YM, Shao CS, Zhang H C, et al (2006) Effects of Cryptoporus polysaccharide on rat allergic rhinitis associated with inhibiting eotaxin mRNA expression. J Ethnopharmacol 107: 424–430. 16765544 10.1016/j.jep.2006.03.040

[26] Gao L, Zhang WW, Sun YP, Yang Q, Ren J, et al (2013) *Cryptoporus volvatus* extract inhibits porcine reproductive and respiratory syndrome virus (PRRSV) *in vitro* and *in vivo* . PLoS One 8: e63767. 23704937 10.1371/journal.pone.0063767 PMC3660591

[27] Jiang Y, Wong JH, Fu M, Ng TB, Liu ZK, et al (2011) Isolation of adenosine, iso-sinensetin and dimethylguanosine with antioxidant and HIV-1 protease inhibiting activities from fruiting bodies of *Cordyceps militarsi* . Phytomedicine 100: 1–5. 10.1016/j.phymed.2010.04.010 20576416

[28] Kim HS, Kwang J, Yoon IJ, Joo HS, Frey ML (1993) Enhanced replication of porcine reproductive and respiratory syndrome (PRRS) virus in a homogeneous subpopulation of MA-104 cell line. Arch Virol 133: 477–483. 8257302 10.1007/BF01313785

[29] Krah DL (1991) A simplified multiwell plate assay for the measurement of hepatitis A virus infectivity. Biologicals 19: 223–227. 1659431 10.1016/1045-1056(91)90039-m

[30] Rowland RRR, Robinson B, Stefanick J, Kim TS, Guanghua L, et al (2001) Inhibition of porcine reproductive and respiratory syndrome virus by interferon-gamma and recovery of virus replication with 2-aminopurine. Arch Virol 146: 539–555. 11338389 10.1007/s007050170161 PMC7087212

[31] Patel D, Opriessnig T, Stein DA, Halbur PG, Meng XJ, et al (2008) Peptide-conjugated morpholino oligomers inhibit porcine reproductive and respiratory syndrome virus replication. Antiviral Res 77: 95–107. 17959259 10.1016/j.antiviral.2007.09.002 PMC7114306

[32] Han X, Fan S, Patel D, Zhang YJ (2009) Enhanced inhibition of porcine reproductive and respiratory syndrome virus replication by combination of morpholino oligomers. Antiviral Res 82: 59–66. 19428596 10.1016/j.antiviral.2009.01.009 PMC7114178

[33] Livak KJ, Schmittgen TD (2001) Analysis of relative gene expression data using real-time quantitative PCR and the 2(-Delta Delta C(T)) method. Methods 25: 402–408. 11846609 10.1006/meth.2001.1262

[34] Wang C R, Qiao W T, Zhang Y N, Liu F (2013) Effects of adenosine extract from *Pholiota adiposa* (Fr.) Quel on mRNA expressions of superoxide dismutase and immunomodulatory cytokines. Molecules 18: 1775–1782. 23434863 10.3390/molecules18021775 PMC6270628

[35] Cabrera GM, Roberti MJ, Wright JE, Seldes AM (2002) Cryptoporic and isocryptoporic acids from the fungal cultures of *Polyporus arcularius* and *P. ciliatus* . Phytochemistry 61: 189–193. 12169314 10.1016/s0031-9422(02)00221-2

[36] Narisawa T, Fukaura Y, Kotanagi H, Asakawa Y (1992) Inhibitory effect of cryptoporic acid E, a product from fungus *Cryptoporus volvatus* on colon carcinogesis induced with N-methyl-N-nitrosourea in rats and with 1,2-dimethylhydrazine in mice. Jpn J Cancer Res 83: 830–834. 1399820 10.1111/j.1349-7006.1992.tb01987.x PMC5918951

[37] Asakawa Y, Hashimoto T, Mizuno Y, Tori M, Fukazawa Y (1992) Cryptoporic acids A-G, drimane-type sesquiterpenoid ethers of isocitric acid from the fungus *Cryproporus volvatus* . Phytochemistry 31(2): 579–592.

[38] Jansen BJM, Groot A (2004) Occurrence, biological activity and synthesis of drimane sesquiterpenoids. Nat Prod Rep 21: 449. 15282630 10.1039/b311170a

[39] Srinivas RV, Robbins BL, Connelly MC, Gong YF, Bischofberger N, et al (1993) Metabolism and *in vitro* antiretroviral activities of bis(Pivaloyloxymethyl)prodrugs of acyclic nucleoside phosphonates. Antimicrobial Agents and Chemotherapy 37(10): 2247–2250. 8257154 10.1128/aac.37.10.2247 PMC192261

