# Supplementary figures and images for: A Novel Compound from the Mushroom Cryptoporus volvatus Inhibits Porcine Reproductive and Respiratory Syndrome Virus (PRRSV) In Vitro
Source: PLoS One. 2013 Nov 18;8(11):e79333. doi: 10.1371/journal.pone.0079333 (PMC3832501; doi:10.1371/journal.pone.0079333)

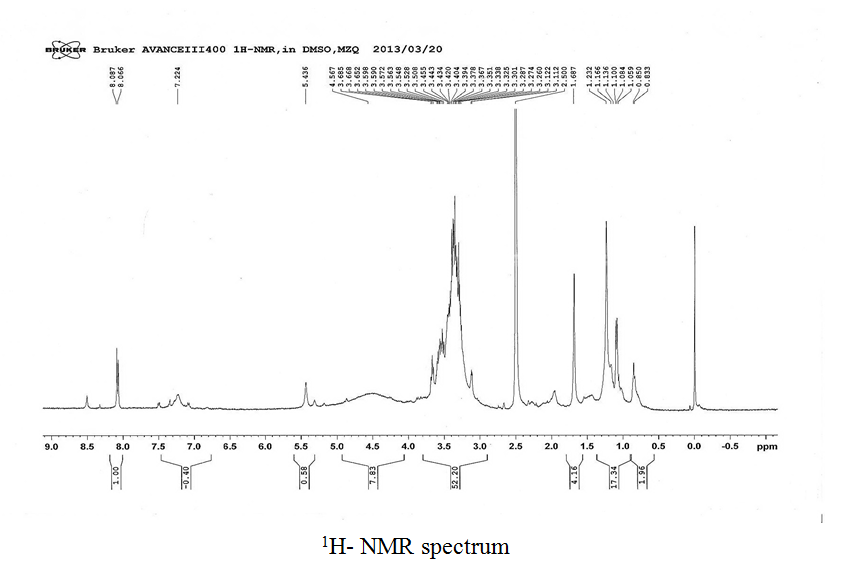

Supplement: Figure S1 — 1H-NMR spectrum. 1H-NMR spectra were acquired with the use of a BRUKER AVANCEIII-400 spectrometer. (TIF) [file pone.0079333.s001.tif]

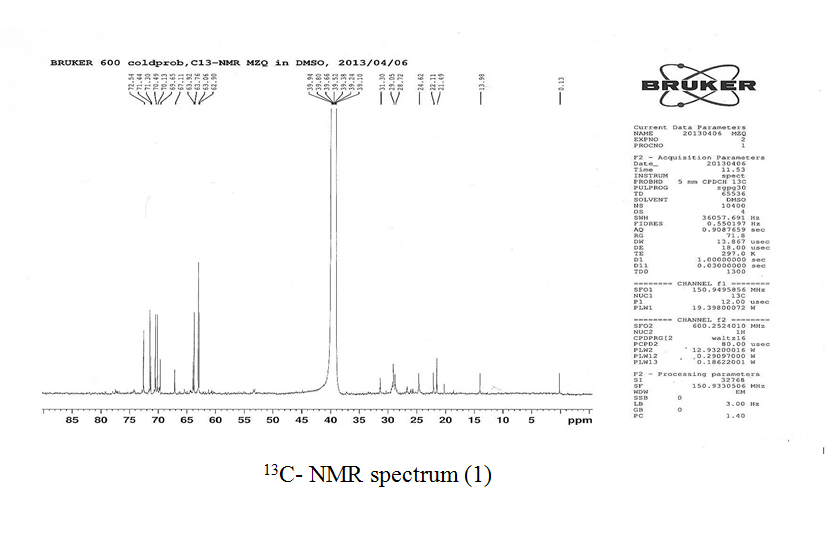

Supplement: Figure S2 — 13C-NMR spectrum (1). 13C-NMR spectra were acquired with the use of a BRUKER AVANCEIII-600 spectrometer. The peaks at δ60–70 ppm showed the presence of polyol fragment containing 11 or 12 carbon atoms. (TIF) [file pone.0079333.s002.tif]

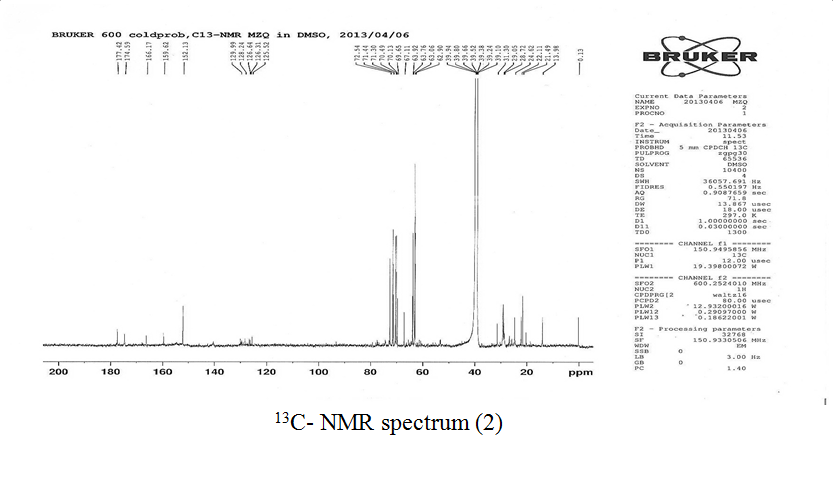

Supplement: Figure S3 — 13C-NMR spectrum (2). 13C-NMR spectra were acquired with the use of a BRUKER AVANCEIII-600 spectrometer. The peak at δ90–110 ppm in the 13C-NMR spectrum of CM-H-L-5 indicated the absence of sugar fragments. (TIF) [file pone.0079333.s003.tif]
